# Electrophoretic Deposition of Chitosan/h-BN and Chitosan/h-BN/TiO_2_ Composite Coatings on Stainless Steel (316L) Substrates

**DOI:** 10.3390/ma7031814

**Published:** 2014-03-04

**Authors:** Namir S. Raddaha, Luis Cordero-Arias, Sandra Cabanas-Polo, Sannakaisa Virtanen, Judith A. Roether, Aldo R. Boccaccini

**Affiliations:** 1 Institute of Biomaterials, Department of Materials Science and Engineering, University of Erlangen-Nuremberg, Cauerstrasse 6, 91058 Erlangen, Germany; E-Mails: namir.jackoub@studium.uni-erlangen.de (N.S.R.); luis.cordero@studium.uni-erlangen.de (L.C.-A.); Sandra.Cabanas@ww.uni-erlangen.de (S.C.-P.); 2 Institute of Surface Science and Corrosion, Department of Materials Science and Engineering, University of Erlangen-Nuremberg, Martensstrasse 7, 91058 Erlangen, Germany; E-Mail: virtanen@ww.uni-erlangen.de; 3 Institute of Polymer Materials, Department of Materials Science and Engineering, University of Erlangen-Nuremberg, Martensstrasse 7, 91058 Erlangen, Germany; E-Mail: judith.roether@ww.uni-erlangen.de

**Keywords:** electrophoretic deposition, nanocomposites, chitosan, TiO_2_

## Abstract

This article presents the results of an experimental investigation designed to deposit chitosan/hexagonal boron nitride (h-BN) and chitosan/h-BN/titania (TiO_2_) composites on SS316L substrates using electrophoretic deposition (EPD) for potential antibacterial applications. The influence of EPD parameters (voltage and deposition time) and relative concentrations of chitosan, h-BN and TiO_2_ in suspension on deposition yield was studied. The composition and structure of deposited coatings were investigated by FTIR, XRD and SEM. It was observed that h-BN and TiO_2_ particles were dispersed in the chitosan matrix through simultaneous deposition. The adhesion between the electrophoretic coatings and the stainless steel substrates was tested by using tape test technique, and the results showed that the adhesion strength corresponded to 3B and 4B classes. Corrosion resistance was evaluated by electrochemical polarization curves, indicating enhanced corrosion resistance of the chitosan/h-BN/TiO_2_ and chitosan/h-BN coatings compared to the bare stainless steel substrate. In order to investigate the *in-vitro* inorganic bioactivity, coatings were immersed in simulated body fluid (SBF) for 28 days. FTIR and XRD results showed no formation of hydroxyapatite on the surface of chitosan/h-BN/TiO_2_ and chitosan/h-BN coatings, which are therefore non bioactive but potentially useful as antibacterial coatings.

## 1. Introduction

Combinations of polymers and ceramic components can be applied to form organic-inorganic soft composite coatings [1–3]. These composite coatings with tailored stiffness can find applications in several industrial sectors as well as in medicine. For example, soft composite coatings provide better connection between rigid metallic implants and vascularized bone tissue [1,3,4]. One convenient method to produce such coatings is electrophoretic deposition (EPD) [1,2]. In the EPD process, the charge particles or molecules in aqueous or organic suspensions are moved to and deposited onto an oppositely charged electrode under the action of an applied electric field [5–7]. For attaining a uniform particle packing structure of electrophoretic deposits suitable stabilization of the suspensions is required, which depends on the amount of surfactant used, suspension concentration, pH, and conductivity [8]. EPD is advantageous because it offers the possibility of coating substrates of complex shape, accurate control of coating thickness, and simple equipment is required [5,9].

Chitosan is an interesting polymer that has been widely used to produce a variety of coatings in combination with EPD [1,10,11]. Chitosan is a cationic polysaccharide that has been used for biocompatible coatings and drug delivery [12,13]. Due to its biodegradability, biocompatibility,non-toxicity and antibacterial properties, chitosan has attracted much attention for a wide variety of biomedical applications [13,14] and for food packaging [15]. Also, chitosan has been used as a stabilizing agent in AgNCs-chitosan hybrid nanocomposites [16]. The feasibility of cationic EPD of chitosan has been shown in previous studies [13,17].

Hexagonal boron nitride (h-BN) has very similar structure to graphite [18,19]. h-BN presents a layered structure with many unique engineering properties and it has been also investigated as a dental cement and in cosmetics [20,21]. Studies on BN and non-biodegradable polymer composites, such as polyaniline, polystyrene, and copolymer of vinylidene chloride and acrylonitride, have justified boron nitride’s role for improving the mechanical and optical properties of the final composites [22–24].h-BN has been also used in combination with hydroxyapatite exhibiting a strong bond in the HA matrix and leading to grain size refinement [20]. In addition, boron nitride in the form of nanotubes (BNNTs) has been investigated to induce apatite formation in simulated body fluid (SBF) environment for periods of 7, 14 and 28 days [25]. It has been also shown that BNNTs are non-cytotoxic to osteoblasts and macrophages, which are relevant cell types for orthopedic applications [26]. Chen *et al*. [27] reported that BNNTs do not inhibit cell proliferation even after 4 days, and BNNTs were found to be non-cytotoxic to human embryonic kidney cells (HEK-293). h-BN has an advantage for the protection of biospecies against overheating and damage because of its low absorptivity of light, which makesh-BN nanomaterials potentially attractive candidates for biological applications [28,29].

A candidate material for developing protective coatings on metallic substrates is titanium dioxide [5,8]. Being a biocompatible ceramic, titania is used for coatings of metal implants to achieve anti-bacterial effect or corrosion resistance, as well as high biocompatibility [30–32]. The EPD technique has been used extensively to produce TiO_2_ layers [33,34]. Achieving a suitable TiO_2_ dispersion is a key requisite for obtaining good-quality films by EPD since the adequate dispersion of nano-sized TiO_2_ particles in organic or aqueous solvents will determine the final deposit microstructure [5,9]. Different solvents such as acetylaceton and acetone have been used to carry out EPD of TiO_2_ nanoparticles [1,35,36], but also water and water-ethanol mixtures have been used in a more limited scale [37,38].

Herein, we propose the fabrication, for the first time, of chitosan/h-BN/TiO_2_ coating by EPD, as well as a chitosan/h-BN coating for comparable purposes. h-BN was selected to improve the mechanical properties and to improve the integration with TiO_2_ nanoparticles in the coatings. The aim of this research is also to increase the knowledge of chitosan based coatings obtained by electrophoretic deposition which are potentially attractive as antibacterial coatings. Chitosan/h-BN and chitosan/h-BN/TiO_2_ coatings were characterized by XRD, FTIR and SEM. The capability of the coatings to form HA on the surface was studied by *in-vitro* bioactivity tests in simulated body fluid to assess the potential suitability of the coatings as bone contacting materials.

## 2. Results and Discussion

Chitosan is insoluble in water and organic solvents. However, protonated chitosan can be dissolved in water-ethanol mixtures at low pH. Under this condition, the amine groups of chitosan are protonated, according to reaction (1) [39]:

$${\text{Chit-NH}}_{\text{2}}+H_{3}O^{\text{+}}\to {\text{ Chit-NH}}_{\text{3}}{}^{+}+H_{2}O$$ (1)

The EPD of chitosan has been described previously [13]. An electric field provides electrophoretic motion of the positively charged chitosan macromolecules which move towards the cathode. The reactions that occur on the cathode electrode (Equations (2) and (3)) generate basic conditions at the electrode surface [6,39]:

$$2H_{\text{2}}O+2e^{-}\to H_{2}+2{\mathrm{OH}}^{-}$$ (2)

$$O_{2}+2H_{\text{2}}O+4e^{-}\to 4{\mathrm{OH}}^{-}$$ (3)

As a consequence of the pH increase at the cathode surface due to electrochemical decomposition of water, chitosan loses its charge and forms an insoluble deposit [39]:

$$\text{Chit}-{\mathrm{NH}}_{3}+{\mathrm{OH}}^{-}\to \text{Chit}-{\mathrm{NH}}_{2}+H_{\text{2}}O$$ (4)

In this work, composite films were prepared from suspensions of h-BN and of both TiO_2_ and h-BN in chitosan solution.

The presence of chitosan in the system can provide the necessary positive charge to perform EPD and it contributes to the suspension stabilization for EPD of h-BN and TiO_2_. The chitosan can be incorporated in chitosan-h-BN and chitosan-h-BN-TiO_2_ mixtures by two different mechanisms:

- Chitosan adsorbed on the TiO_2_ nanoparticles and/or on h-BN micro particles.
- Non-adsorbed chitosan present in the bulk of the suspension and incorporated in the deposit by direct EPD.

The zeta potential values of C-hBN1, C-hBN2 and C-Ti-hBN suspensions used for EPD experiments were found to be 35 ± 10, 37 ± 11 and 41 ± 9 mV, respectively. As it can be observed, all suspensions present positive values of zeta potential at the working pH of 3.5, which also predicts a cathodic deposition of the particles. Therefore, the cathodic deposition of chitosan, TiO_2_ and h-BN can be combined to form composite films.

**Figure 1.**
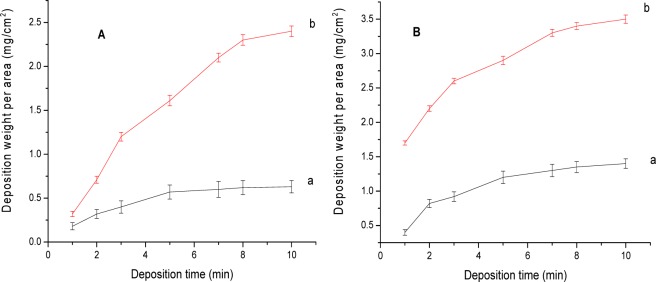
Deposit weight normalized to covered area *vs*. deposition time for the deposits prepared from (**A**) C-hBN2 and (**B**) C-Ti-hBN at (a) 10 V (b) 30 V. Three different samples were prepared for each deposition time and the final results were averaged. Error bars refer to the standard deviation.

After a trial-and-error sequence of experiments, the optimum voltages (10 and 30 V) and deposition time (from 1 to 10 min) were chosen. Figure 1 shows a continuous increase in the deposition weight with increasing deposition time for C-hBN2 (Figure 1A) and C-Ti-hBN (Figure 1B) suspensions. Three different samples were prepared for each deposition time and the final results were averaged (error bars in Figure 1 refer to the standard deviation). The deposition yield measurements were found to be repeatable with an error below 8% and 7% for C-hBN2 and C-Ti-hBN, respectively. It is observed that the slope of the curves decreased with increasing deposition time due to the formation of an electrically insulating film which decreases the voltage drop in the bulk of the suspensions. Increasing the voltage from 10 to 30 V (curves a and b in Figure 1A,B, respectively) resulted in a higher deposition yield which is in agreement with the prediction of Hamaker’s equation [40]:

$$M=\mu EtSC_{S}$$ (5)

where *M* is the mass deposition, μ is the particle electrophoretic mobility, *E* is the electric field, *t* is the deposition time, *S* is the surface area of the electrode, and *C*_s_ is the concentration of colloidal particles in suspension. Keeping μ, *t*, *S*, *C*_s_ constant in each case, then the Hamaker equation can be written as:

M=KE (6)

where K is constant, then showing the proportional relation between yield deposition and applied electric field for a fixed deposition time.

### 2.1. Composition and Microstructure of Coatings

For the further characterization of the coatings, those coatings obtained at 10 V for a deposition time of 5 min were selected given that at this relatively short deposition time, a suitable deposition yield had been obtained (see Figure 1A,B).

**Figure 2.**
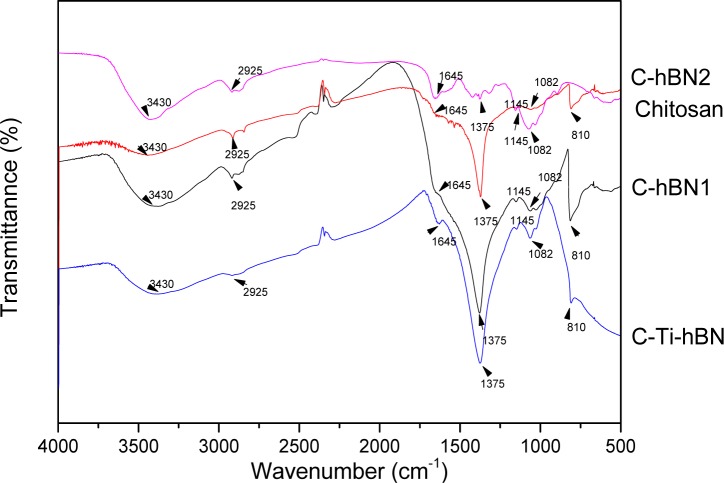
FTIR spectra of C-hBN1, C-hBN2 and C-Ti-hBN coated samples prepared by EPD using 10 V and 5 min. Chitosan powder was used as a reference.

FTIR analyses for coated samples were carried out to investigate the interaction between the chitosan matrix, h-BN and TiO_2_ particles. Three coatings of each system were used for the measurements and a chitosan reference was also analyzed. FTIR spectra of C-hBN1, C-hBN2 andC-Ti-hBN2 are shown in Figure 2.

In C-hBN1 (chitosan-1 g/L h-BN), C-hBN2 (chitosan-2 g/L h-BN) and C-Ti-hBN (chitosan-2 g/Lh-BN-2 g/L TiO_2_) spectra, the characteristic C-O and C-H stretching vibrations of the chitosan molecules appear at 1082 cm^−1^ [18] and 2925 cm^−1^ [1], respectively. The peaks at 1645 cm^−1^ and 3430 cm^−1^ are assigned to the N-H bending of the amines groups and the O-H stretching vibration of the chitosan molecule [1,41–45], respectively. The C-O-C symmetric stretching vibration can be seen at 1145 cm^−1^ [18] and the symmetric deformation of CH_3_ group appears at 1375 cm^−1^ [1]. The BN stretching vibration appears at 810 cm^−1^ [18]. All these peaks indicate that both, chitosan and h-BN are incorporated in the coating. In addition, there is a broad band that spreads below 800 cm^−1^ which, according to the literature [1,46–48], indicates the presence of titania in the coating.

**Figure 3.**
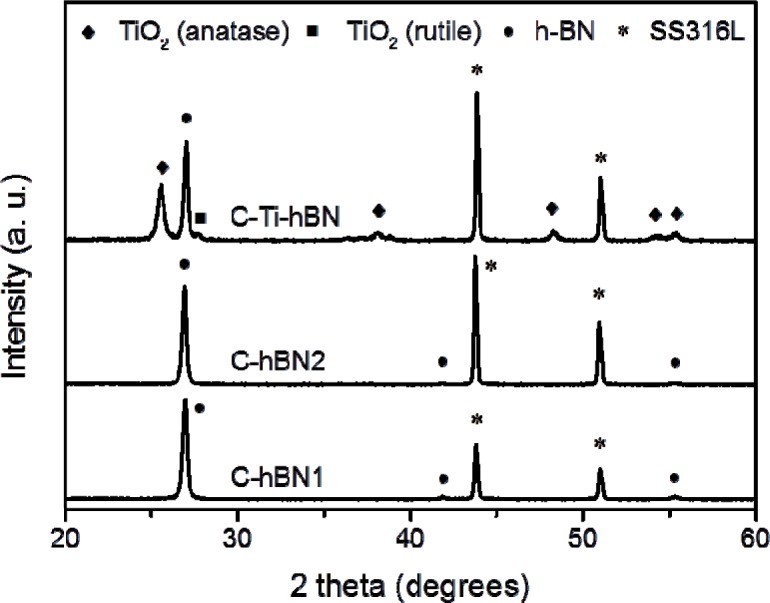
XRD pattern of sample C-hBN1, C-hBN2, C-Ti-hBN prepared by EPD using 10 V and 5 min.

The XRD patterns of C-hBN1, C-hBN2 and C-Ti-hBN coatings are shown in Figure 3. As it can be observed, the peaks corresponding to h-BN appear in all coatings while for C-Ti-hBN, the titania peaks are present. All peaks were indexed using JCPDS files 01-085-1068 for boron nitride also00-002-0406, 01-078-2486 for anatase TiO_2_ and 00-021-1276 for rutile TiO_2_. The identification of both h-BN and titania in the XRD spectra corroborates the presence of both materials in the coatings as was first indicated by FTIR test.

**Figure 4.**
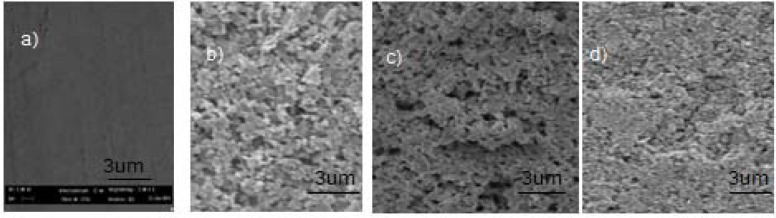

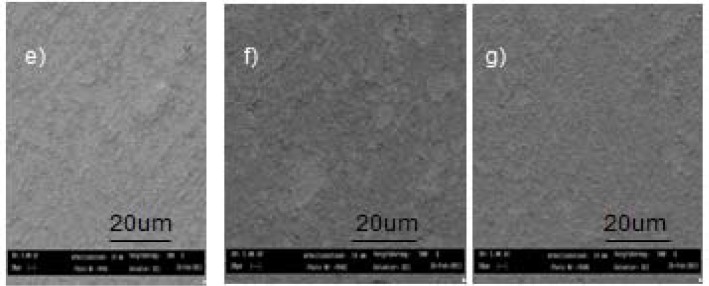
SEM images of (a) non-coated SS substrate and of (b,e) composite films prepared from C-hBN1 suspension; (c,f) C-hBN2 suspension and (d,g) C-Ti-hBN suspension at EPD conditions: 10 V and 5 min.

Figure 4 shows SEM images of C-hBN1 (Figure 4b,e), C-hBN2 (Figure 4c,f) and C-Ti-hBN (Figure 4d,g) coatings obtained using 10 V and 5 min as EPD conditions. An image of the SS substrate (Figure 4a) is also shown for comparison purpose. Images taken at low magnification (Figure 4e–g) show that the films are continuous and crack-free, while the images taken at high magnification(Figure 4b–d) indicate that the films are relatively dense, although some porosity can be observed. No significant qualitative differences are seen in the microstructure of the coatings when the h-BN concentration is increased from 1 g/L (sample C-hBN1) to 2 g/L (sample C-hBN2) as revealed when comparing Figure 4b,c, respectively. Regarding the porosity, it can be seen that the sample obtained with titania (C-Ti-hBN) seem to be less porous than the other ones. This could be due to the smaller particle size of the titania which enables it to fill gaps that have been created between the h-BN particles and, therefore, decreasing the porosity.

**Figure 5.**
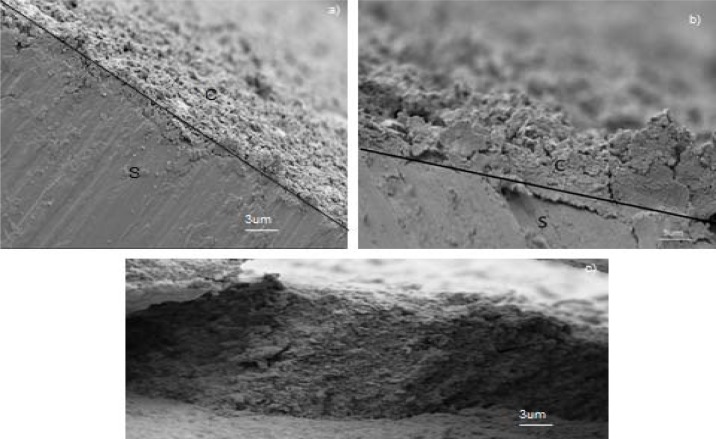
SEM images showing the cross-sections of composite films prepared from suspension (**a**) C-hBN1; (**b**) C-hBN2; (**c**) C-Ti-hBN at 10 V and 5 min. The substrate is indicated by “S” and the coatings by “C” in the images.

SEM images of the cross sections of C-hBN1, C-hBN2 and C-Ti-hBN samples (Figure 5a–c, respectively) show that films were fairly uniform although for sample C-hBN2 (Figure 5b) the thickness seems to be distorted, possibly due to the machining process for SEM observations. In Figure 5a,b, the coatings (denoted by C) are seen to cover the substrate (denoted by S) to a large extent. The thickness achieved was around 2 μm for sample C-hBN1 and varied from 0 to 4 μm when the concentration of h-BN in chitosan changed to 2 g/L (sample C-hBN2). However, a maximum thickness of ~12 μm was achieved for C-Ti-hBN which correlates with the higher yield measured for this sample, when titania is also incorporated in the coating (as shown in Figure 5c).

**Figure 6.**
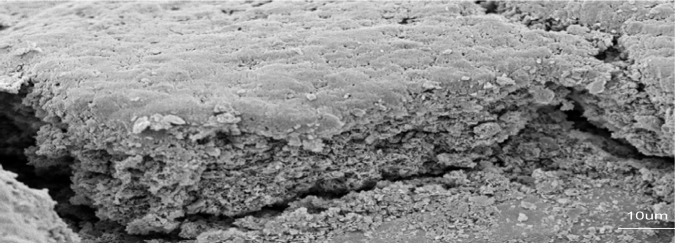
SEM image of the cross-section of sample C-hBN2 prepared at 30 V and 5 min.

In Figure 6, when the voltage is increased up to 30 V for C-hBN2 sample (keeping the time constant at 5 min), a higher amount of material on the substrate is observed and thicker coatings of around ~15 μm are obtained as expected from Equation (6).

It can also be observed that microcracks are formed on the coated surface. This is due to the formation of a relatively thick layer, which undergoes rearrangement upon contraction during drying, where the shrinkage of the deposit coating could be substantially different from the substrate and as a result, tensile/compressive stresses are developed in the coating and relieved by the formation and propagation of cracks. If the layers were thinner, the formation of cracks would be less accused since the ratio h-BN/chitosan would be lower, and also considering that the metallic substrate would have adhered better to the thinner h- BN layer embedded in chitosan.

### 2.2. Corrosion Behavior

**Figure 7.**
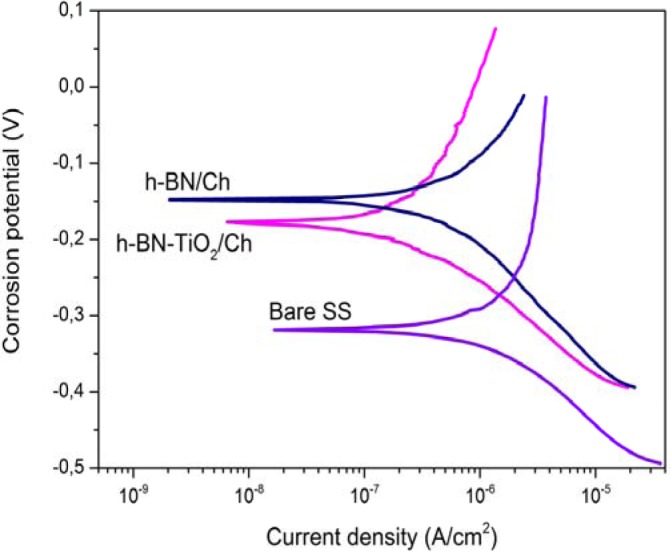
Polarization curves in DMEM for bare SS 316L and SS coated with a film prepared from C-Ti-hBN suspension and C-hBN2 suspension at 37 °C.

Corrosion resistance of metal substrates used in biological environments is an important issue that gives an indication of the biocompatibility of the materials, as corrosion products must be minimized. Polarization curves for C-Ti-hBN, C-hBN2 and bare stainless steel substrate (SS) are shown in Figure 7. The results show a lower corrosion current for all coated systems compared with the uncoated metallic substrate, being proof of the corrosion protective properties of these coatings. It can be also observed that the presence of TiO_2_ leads to further reduction of the corrosion current density, indicating a better corrosion protection by this coating type. This effect may be attributed to the small particle size of the titania powder that could be covering possible open spaces or pores of the coating where the DMEM can penetrate being in direct contact with the substrate. That is, the titania nanoparticles could be filling the gaps between h-BN particles and, therefore, create a more continuous coating that provides better corrosion protection, as was explained above when discussing the coating’s microstructure. The electrochemical results obtained for C-Ti-hBN are in agreement with similar organic-inorganic composite coatings via EPD, previously obtained and tested under the same experimental conditions [1,49].

### 2.3. Adhesion of Coatings to Substrate

**Figure 8.**
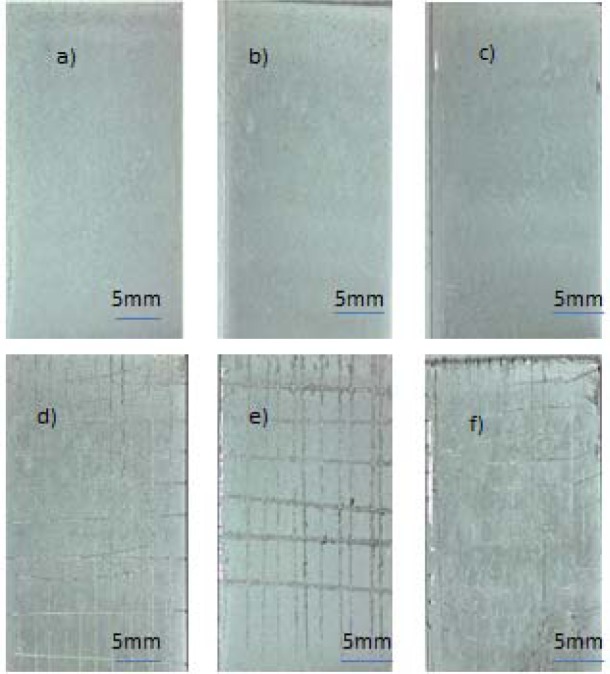
Typical optical images of coatings before and after adhesive tests for (**a**,**d**) C-Ti-hBN; (**b**,**e**) C-hBN2; (**c**,**f**) C-hBN1 suspensions obtained using 10 V and 5 min.

The adhesion strength between the coatings and the SS 316L substrates was assessed qualitatively by the adhesive tape test. Results of the adhesion strength for all composite coatings according to the ASTM D3359-B standard showed that the adhesion strength corresponded to class 3B for C-hBN1 and C-hBN2, and class 4B for C-Ti-hBN (Figure 8). The adhesion for C-Ti-hBN is better than in C-hBN1 and C-hBN2 because the dispersion of titania nano particles in the matrix gives more surface areathat allows chitosan to penetrate between micro (h-BN) and nano-particles (TiO_2_), which results in improved attachment between the coating layer and substrate.

### 2.4. Bioactivity Study

**Figure 9.**
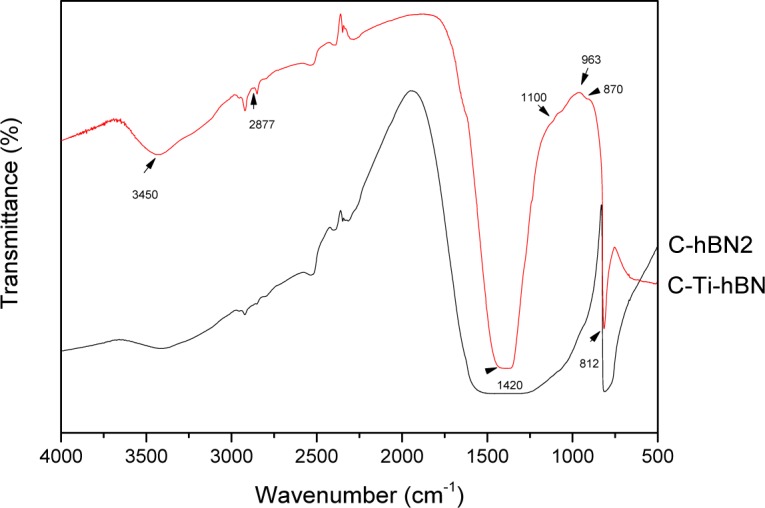
FTIR spectroscopy patterns of coatings after 28 days’ immersion in simulated body fluid (SBF) for C-hBN2 and C-Ti-hBN.

**Figure 10.**
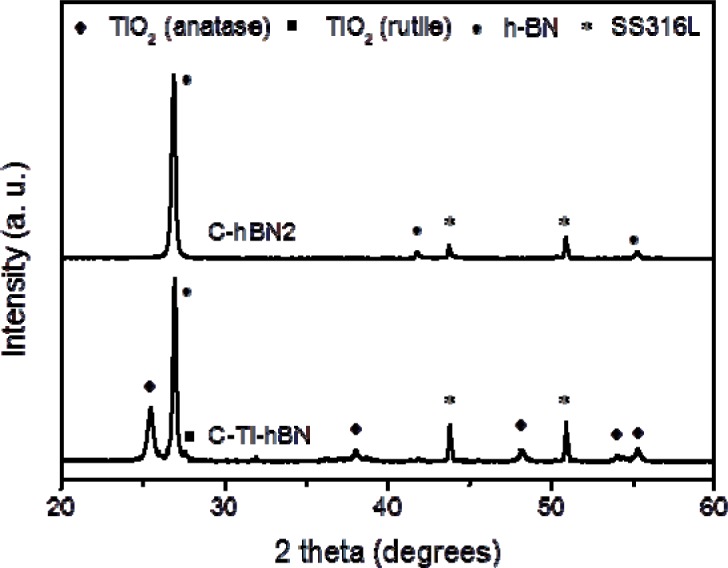
XRD patterns of C-hBN2 and C-Ti-hBN coatings after 28 days’ immersion in SBF.

The formation of HA in SBF is usually considered the marker of the bioactive character of materials used as bone replacement and as orthopedic coatings [50–52]. The hydroxyapatiteforming ability of the coatings was investigated by FTIR and XRD after 28 days of immersion in SBF (Figures 9 and 10), respectively.

In FTIR spectra, in order to confirm that HA was formed, a double peak at 560–600 cm^−1^ and a broad peak at 1000–1100 cm^−1^ should be present in the spectrum [53]. For C-Ti-hBN and C-hBN samples, those characteristic peaks are not obviously present; therefore, there is no evidence of formation of HA and it can be stated that the coatings are not bioactive. The same conclusion can be drawn from the XRD results (Figure 10), *i.e.*, it is not possible to confirm the formation of HA on the coatings after immersion in SBF for 28 days.

## 3. Experimental Section

Chitosan powder with deacetylation degree of about 85% was purchased from Sigma Aldrich (Taufkirchen bei München, Germany) and two different sources of acetic acid with the same analytical properties were purchased from Sigma Aldrich (Taufkirchen bei München, Germany) and VWR Chemicals. The hexagonal BN powder (h-BN) was of average particle size 2.1 μm. Titanium dioxide (TiO_2_) grade P25, which is a very fine powder with mean particle size of 21 nm, was obtained from Evonik industries. Ethanol was purchased from Merck KGaA (Darmstadt, Germany).

Chitosan/h-BN and chitosan/h-BN/TiO_2_ composite films were prepared from suspensions containing variable h-BN loadings and a fixed chitosan concentration of 0.5 g/L. Chitosan solution was prepared by dissolving chitosan powder in 1 vol% aqueous acetic acid solution. Three different suspensions were prepared, the first two with different h-BN contents (1 and 2 g/L) (labeled C-hBN1 and C-hBN2, respectively) and the third one with 2 g/L h-BN and 2 g/L TiO_2_ (labeled C-Ti-hBN). Suspensions were prepared by adding the h-BN and TiO_2_ to a 0.5 g/L chitosan solution. A mixture of water and ethanol with 17 vol% of distilled water was used.

After preparation of the above suspensions (C-hBN1, C-hBN2, C-Ti-hBN) they were stirred magnetically for 24 h and then passed through the ultrasound bath (Sonorex 120 W/80 kHz from Bandelin electronics-Germany) for 15 min. pH-indicator strips (non-bleading) from Merk KG, Darmstadt, Germany, were used to measure the pH of the suspensions. AISI 316L stainless steel electrodes (30 mm × 15 mm × 0.2 mm) were utilized as deposition substrates and counter electrodes in the EPD cell. The electrodes were ultrasonically washed in ethanol for 10 min. EPD was performed under constant voltage conditions. Two different voltages (10 and 30 V) and different periods of deposition time (1, 2, 3, 5, 7, 8 and 10 min) were studied. The distance between the electrodes was kept constant at 15 mm. A constant electric voltage was applied by a Telemeter electronic GmbH TTi Ex 752 M 75 V/150 V 300 W power supply and the current through the suspension during EPD was recorded by a 1906 Computing Multimeter from Thurlby-Thandar instruments LTD (Huntingdon, England). EPD of C-hBN1, C-hBN2 and C-Ti-hBN was carried out without stirring. Deposit weights were obtained by weighing the substrates before and after the deposition process followed by drying at room temperature for 24 h.

In order to ensure the reproducibility and homogeneity of the coatings, the stability of the C-hBN1, C-hBN2 and C-Ti-hBN suspensions was studied in terms of zeta potential, measured by laser Doppler velocimetry (LDV) technique, using a Zetasizer nano ZS equipment (Malvern Instruments, Malvern, UK). The LDV method measures the electrophoretic mobility of the particles and, after applying Henry’s equation, transforms that value into zeta potential. Suspensions were diluted down to 0.1 g/L as a requirement to obtain reliable measurements.

X-ray diffraction measurements of the coatings were performed to determine the composition ofthe coatings. The diffractograms were obtained using a X-ray diffractometer (XRD) (D500 Siemens, Siemens, München, Germany) CuKα 1.2 secondary-Monochromator with 0.02 degree as a 2θ step and operated at 30 KV. The surface microstructures of the deposited coatings were investigated using a LEO-435 VP scanning electron microscope (SEM) (Leo Scanning Electron Microscopes Ltd., Cambridge, England). The SEM specimens were coated with an alloy of gold and palladium to improve surface conductivity for SEM observations. Fourier transform infrared (FTIR) spectroscopy (Nicolet 6700, Thermo Scientific, Waltham, MA, USA) measurements were performed to record spectra of the coated samples in the wave number range of 400–4000 cm^−1^.

The adhesion between coatings and substrates was tested by the tape test according to ASTM standards [54] using Elcometer 107 cross hatch cutter (Manchester, UK). The tests were carried out by using a cutting tool and this was placed on the substrate at 90 degrees to make a series of parallel cuts (approximately 20 mm long) by pressing down and pulling the tool towards the operator. A suitable length of adhesive tape was selected and centered over the lattice. After a short time (about 90 s), the tape was removed at an angle of 180 degrees to the coating surface. At the end, the lattice of cuts was compared with ASTM standard. The ASTM standard values for adhesion strength assigns six quality classes, namely 5B for 0% removal, 4B for less than 5% removal, 3B for 5%–15% removal, 2B for 15%–35% removal and 0B for more than 65% removal [54,55].

An electrochemical evaluation in cell culture medium was performed to investigate the corrosion behavior of C-hBN2 and C-Ti-hBN as well as of the uncoated SS 316L substrates. Polarization curves were obtained using a potentiostat/galvanostat (Autolab PGSTAT 30). The samples were immersedin 100 mL of Dulbecco’s MEM (Biochrom) at 37 °C and the solution was not stirred during the experiment. A conventional three electrode system was used, where a platinum foil served as counter electrode and Ag/AgCl (3 mol/L KCl) was used as reference electrode. The analysis was carried out using an O-ring cell with an exposed sample area of 0.78 cm^2^ with a potential sweep rate of 1 mV/s.

An *in-vitro* bioactivity assessment, was carried out in simulated body fluid (SBF) prepared according to literature [50]. Each coated sample was immersed in 50 mL of (SBF) and kept at 37 °C for 28 days. The SBF was changed every three days to keep constant the ionic concentration. After immersion in SBF, the substrates were dried at room temperature. FTIR and XRD analyses were performed on the samples after immersion to analyze possible hydroxyapatite (HA) formation.

## 4. Conclusions

A novel family of h-BN/chitosan and h-BN/TiO_2_/chitosan coatings was successfully obtained on stainless steel 316L substrates by EPD. The method was based on the electrophoresis of protonated polymer (chitosan) molecules in acidic solution, base generation at the cathode surface, charge neutralization and deposition of an insoluble polymer film.

Following the successful EPD of h-BN and h-BN/TiO_2_, the deposition yield, composition, morphology, and film thickness were measured. It was shown that the latter could be controlled by varying deposition parameters such as the deposition voltage. Film thicknesses of 0–4 μm were observed for C-hBN1 and C-hBN2 at 10 V while the thickness for C-Ti-hBN film could be increased from ~12 μm at 10 V to ~15 μm at 30 V when using a deposition time of 5 min.

Porosity was decreased in C-Ti-hBN film comparing with C-hBN1 and C-hBN2 films, due to the smaller particle size of the titania which enables filling the gaps between h-BN particles.

Analysis of the film showed suitable adhesion strength to the substrate for chitosan/hBN and chitosan/hBN/TiO_2_. Also, enhanced corrosion resistance compared to bare SS substrate before deposition was confirmed showing that chitosan/h-BN/TiO_2_ provides better corrosion protection than chitosan/h-BN coatings. The results of this investigation showed that EPD is a versatile method for the fabrication of chitosan based composite materials. The results confirm that the composite coating would act as a protective layer to provide corrosion protection and also improved adhesion strength. The application of the developed coatings as antibacterial layers is the focus of current studies.
